# Effects of Low-Level Persistent Infection on Maintenance of Immunity by CD4 T Cell Subsets and Th1 Cytokines

**DOI:** 10.1128/iai.00531-22

**Published:** 2023-02-28

**Authors:** Samad A. Ibitokou, Komi Gbedande, Michael M. Opata, Victor H. Carpio, Karis M. Marshall, Robin Stephens

**Affiliations:** a Department of Internal Medicine, Division of Infectious Diseases, University of Texas Medical Branch, Galveston, Texas, USA; b Microbiology and Immunology, University of Texas Medical Branch, Galveston, Texas, USA; University of Pennsylvania

**Keywords:** helper T cell, T-cell immunity, Th1/Th2 responses, apicomplexan parasites, malaria

## Abstract

CD4 T cells are required, along with antibodies, for complete protection from blood-stage infection with Plasmodium spp., which cause malaria. Without continuous exposure, as on emigration of people from endemic areas, protection from malaria decays. As in other persistent infections, low-level Plasmodium chabaudi infection protects the host from reinfection at 2 months postinfection, a phenomenon termed premunition. Premunition is correlated with T cell responses, rather than antibody levels. We previously showed that while both effector T cells (Teff) and memory T cells (Tmem) are present after infection, Teff protect better than Tmem. Here, we studied T cell kinetics post-infection by labeling dividing *Ifng^+^* T cells with 5-bromo-2′-deoxyuridine (BrdU) in infected *Ifng* reporter mice. Large drops in specific T cell numbers and *Ifng*^+^ cells upon clearance of parasites suggest a mechanism for decay of protection. Although protection decays, CD4 Tmem persist, including a highly differentiated CD27^−^ effector memory (Tem) subset that maintains some *Ifng* expression. In addition, pretreatment of chronically infected animals with neutralizing antibody to interferon gamma (IFN-γ) or with clodronate liposomes before reinfection decreases premunition, supporting a role for Th1-type immunity to reinfection. A pulse-chase experiment comparing chronically infected to treated animals showed that recently divided *Ifng^+^* T cells, particularly IFN-γ^+^ TNF^+^ IL-2^−^ T cells, are promoted by persistent infection. These data suggest that low-level persistent infection reduces CD4^+^ Tmem and multifunctional Teff survival, but promotes IFN-γ^+^ TNF^+^ IL-2^−^ T cells and *Ifng^+^* terminally differentiated effector T cells, and prolongs immunity.

## INTRODUCTION

Malaria still accounts for 627,000 deaths globally, with an estimated 77% in children under 5 years of age (1). Morbidity from malaria is so prevalent that it has a measurable detrimental effect on the economic output of developing nations (2). Plasmodium falciparum can last at least 1 year in untreated people (3), and Plasmodium chabaudi infection lasts 2 to 3 months. The late phase of low-level parasitemia does not induce fever or weight loss, suggesting coevolutionary adaptation. Indeed, persistent P. chabaudi infection confers protection from reinfection. Protection during persistent infection is a phenomenon historically termed premunition and is also seen in other persistent infections (4). Constant and persistent exposure protects people also, as those who emigrate out of endemic areas lose protection. Clinical disease and its severity are increased years later when infected again upon return to endemic areas compared to disease in age-mates who do not emigrate (5). The adaptive mechanisms of premunition are not well understood. Plasmodium chabaudi AS blood-stage infection in mice has been well characterized and is quite reproducible (6). A maximum level of parasitemia occurs between days 8 and 10 postinfection (p.i.) and is largely resolved by day 20 p.i. but is followed by variable small recrudescent peaks up to day 75, with levels lasting less than 90 days (7). These levels of parasitemia protect from reinfection for up to 2.5 months after primary infection, as has been documented (7). Therefore, P. chabaudi infection of C57BL/6 mice represents a model of persistent Plasmodium infection that can be used to understand the mechanisms of premunition.

As full protection from reinfection is lost at a time point corresponding to the end of persistence of the primary infection. Understanding the cell types correlated with this protective phase and their decay may help us to design better, longer-lasting vaccines. In humans, Plasmodium-specific B cells and interferon-gamma (IFN-γ)^+^ effector/memory phenotype T cells (Teff/Tmem) accumulate with P. falciparum exposure, correlating with the development of clinical immunity (8, 9). Plasmodium-specific memory B cells have been shown to be stable in humans for up to 16 years (10) and in mice for at least 200 days (11) However, the correlates of protection and survival of Plasmodium-specific memory CD4 T cells are only beginning to be understood (12, 13).

To determine correlates of protection in persistent infection, it is important to understand the kinetics of Th1 cell survival and the effects of persistent infection on cytokine production. Our previous studies demonstrate that P. chabaudi infection produces a mixture of Teff and effector memory T cells (Tem) in the persistent phase (14). We also showed that transferred Teff protect from parasitemia more effectively than Tmem (15). Our work suggests that MSP1-specific transgenic Teff activated by P. chabaudi infection protect immunodeficient mice from P. chabaudi infection much better than Tem. Clearance of persistent P. chabaudi infection at day 30 p.i. with antimalarial drugs reduced the protection of premunition at day 60. However, late treatment reduced Th1 cytokines but not Tem, suggesting that Tem are not responsible for the improved protection of premunition. In the same study, we showed that IFN-γ^+^ TNF^+^ IL-2^−^ CD62L^lo^ cells were correlated with premunition, though we were unable to determine if they were Teff or Tem (14).

Understanding the mechanisms underlying the durability of protection is also critical for children facing cyclic malaria seasons. Our previous studies showed that parasite-specific Teff decay more quickly than Tem upon transfer into uninfected recipients. Therefore, we hypothesized that the decay of protective Teff explains the decay of immunity. Freitas do Rosario et al. showed in mice that it is the decay of P. chabaudi-responsive CD4 T cells, and not the level of parasite-specific antibody, that correlates with the decay of protection from parasitemia upon reinfection that occurs between days 120 and 200 p.i. (11).

Here, in order to elucidate the differential kinetics of effector and memory T cells, we have studied the full physiological T cell response to P. chabaudi over a long follow-up period postinfection. We show that CD4 T cells are detectable in blood after P. chabaudi infection and decay in two waves. A decline of T cell numbers occurs after the first parasitemia peak, while the Tmem numbers and a constant fraction of *Ifng*/*Thy1.1*^+^ T cells in each subset are maintained more stably. Neutralization of IFN-γ or depletion of phagocytic cells before reinfection reduced protection, supporting a role for a Th1-type response in immunity. Studying T cell turnover, we identified Teff maintained by persistent infection that retained *Ifng* reporter expression and showed that eliminating low-level chronicity slightly reduced *Ifng*^+^ Teff. In addition, persistent infection reduced multifunctional IFN-γ^+^ TNF^+^ IL-2^+^ Teff but promoted IFN-γ^+^ TNF^+^ IL-2^−^ T cells. This finding suggests that in persistent infection, cytokine multiproducers are Teff and are specifically reduced by persistent stimulation. Overall, these data highlight the important role of Teff and the Th1 response in maintaining the protection from Plasmodium infection during low-level persistent parasitemia.

## RESULTS

### CD4 effector T cells are detectable in blood in two waves after P. chabaudi infection and decay.

**FIG 1:**
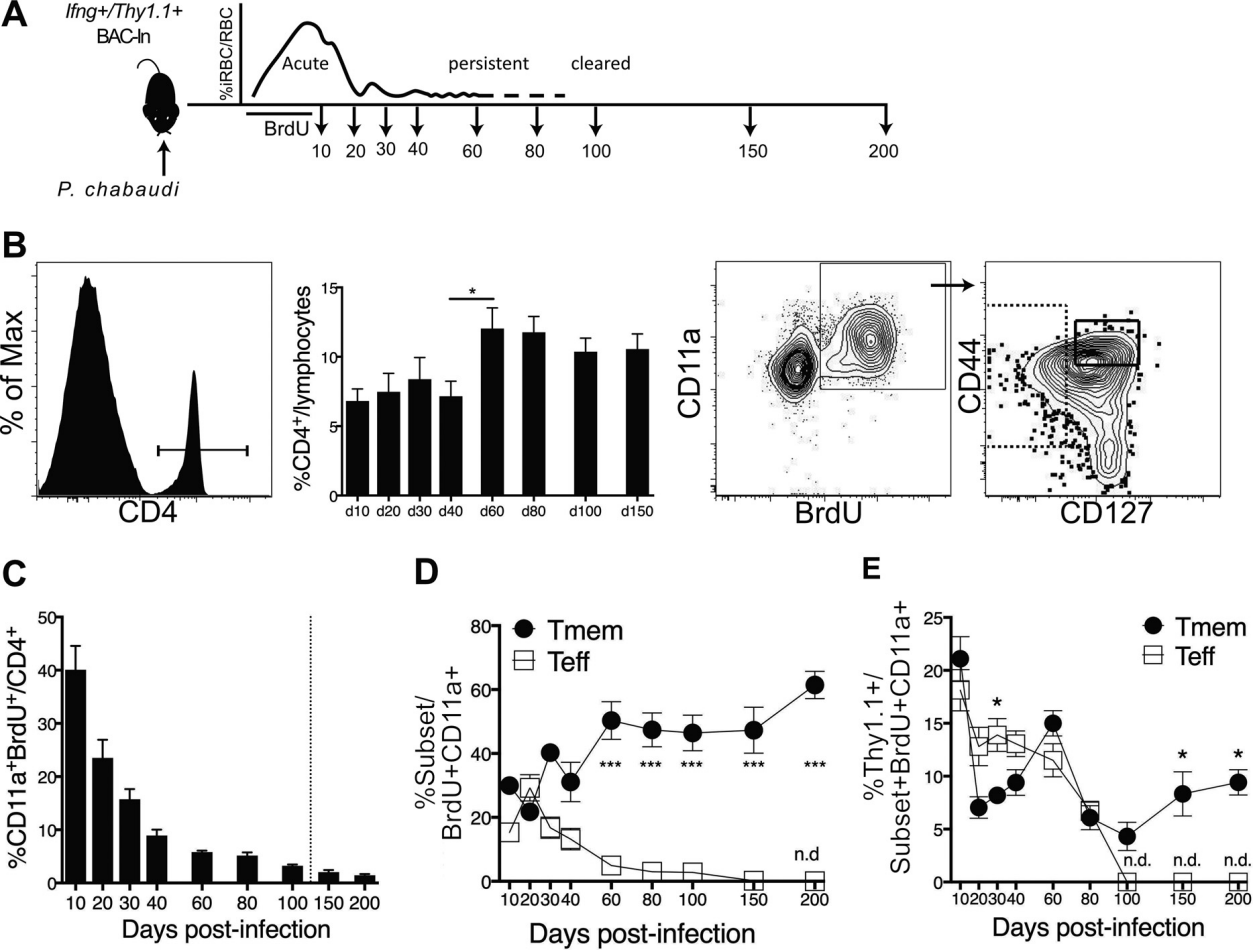
Decay of Teff and survival of Tmem after P. chabaudi infection. *Ifng*/*Thy1.1* BAC-In reporter mice were infected with P. chabaudi and given BrdU from days 3 to 10 p.i. to label dividing cells. Blood was collected at the indicated time points (days 10 to 200 p.i.) to study the decay of Teff and Tmem generated during days 3 to 10 p.i. (A) Schematic representation of the experimental design. (B) Flow cytometry gating strategy showing percentages of CD4^+^ out of blood lymphocytes, CD4^+^ CD11a^+^ BrdU^+^ Teff (CD127^−^), and Tmem (CD44^hi^ CD127^+^). (C to E) Graphs showing percentages of CD11a^+^ BrdU^+^ out of blood CD4 T cells (C), fractions of Teff and Tmem within divided BrdU^+^ CD11a^+^ CD4^+^ T cells (D), and fractions within BrdU^+^ CD11a^+^ Teff and Tmem that were *Ifng*/*Thy1.1*^+^ (E). Data are representative of three experiments with five mice per group. Error bars represent SEM. The Student *t* test was used. *, *P* < 0.05; ***, *P* < 0.001; n.d., none detected.

The course of parasitemia in P. chabaudi infection has been extensively studied. P. chabaudi blood-stage infection of C57BL/6 mice is characterized by a large peak of parasitemia (Fig. 1A) that peaks before the acute symptomatic phase (10 to 14 days p.i.) and is followed by persistent small recrudescent peaks (detectable by blood smear up to day 30 p.i.) that are completely cleared between 2 and 3 months postinfection (7). Increased protection attributable to persistent infection, or premunition, is present through the first 2 months, and a further loss of protection can be measured by day 200 p.i. (11). The only correlate of premunition at day 60 so far is an increase in IFN-γ^+^ TNF^+^ IL-2^−^ CD62L^lo^ T cells in persistently infected mice compared to the level in mice treated with chloroquine at day 30 (12). It is not yet known if these are short-lived effector T cells proliferating in response to persistent infection or effector memory T cells.

To understand the longevity and decay of CD4^+^ *Ifng*^+^ T cells during premunition, IFN-γ reporter mice were infected with P. chabaudi AS. Dividing P. chabaudi-responsive T cells in *Ifng*/*Thy1.1* BAC-In mice were labeled with 5-bromo-2′-deoxyuridine (BrdU) for 7 days, up to and including the peak of T cell expansion on day 9 p.i. (Fig. 1A) (16). CD4 T cells activated by infection were detected in the blood (Fig. 1B) by flow cytometry using antibodies to BrdU and CD11a, which has been shown to be upregulated in response to antigen, but not cytokines (17). As interleukin-7 receptor subunit alpha (IL-7Rα [CD127]) is completely downregulated upon activation of CD4 T cells, we used this as a marker to distinguish Teff from Tmem. Teff in the blood were defined as CD127^int/−^ (intermediate/negative), in contrast to Teff in the spleen, where the CD127^−^ population represents a discreet subset at the peak of infection (14). T cell activation was followed over 200 days using the fraction of CD4 T cells in the blood as a stable denominator to quantify the changes in T cell subsets over time (Fig. 1C). Responsive T cells (CD4^+^ BrdU^+^ CD11a^+^) showed a decay, particularly between days 10 and 20 postinfection, and a slower consistent rate of decline after that. Specifically, only half of the divided T cells remained by day 20 postinfection (p.i.) after T cell contraction, while some Plasmodium-specific T cells remained detectable in the blood for 200 days. The fraction of CD127^int/−^ Teff of total CD11a^+^ BrdU^+^ CD4 in the blood was significantly decreased compared to that of memory T cells by day 60 p.i. (Fig. 1D). Teff, but not Tmem, became undetectable by day 200 p.i., when protection from parasitemia has been shown to decay (K. Gbedande, S. A. Ibitokou, M. L. Ong, M. A. Degli-Esposti, M. G. Brown, and R. Stephens, unpublished data; 11). The decay of *Ifng*^+^ T cells followed different kinetics, with *Ifng*/*Thy1.1^+^* Teff still detectable until day 80 (Fig. 1E). BrdU^+^ Teff contained more *Ifng^+^* cells than Tmem on day 30 p.i, but BrdU^+^ *Ifng*^+^ Teff became undetectable in the blood by day 100. Some *Ifng*^+^ Tmem derived from the peak of infection (5 to 10%) remained *Ifng*^+^, even after parasite clearance.

### A fully differentiated Tem subset can maintain IFN-γ production.

**FIG 2:**
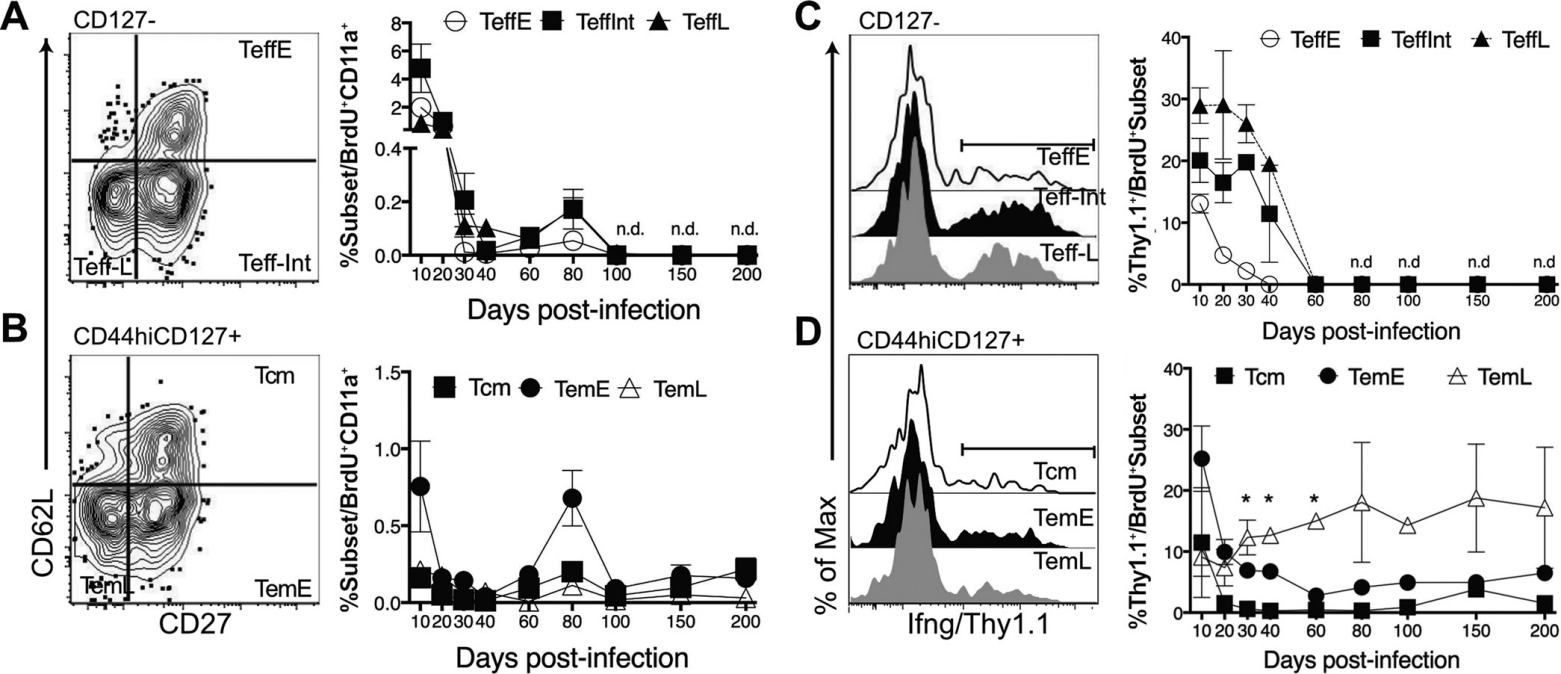
Decay of *Ifng* expression in P. chabaudi infection. *Ifng*/*Thy1.1* BAC-In reporter mice were infected with P. chabaudi and given BrdU from days 3 to 10 p.i. to label dividing cells. Blood was collected (days 10 to 200) to study the decay of labeled cells generated from days 3 to 10 p.i., including their current Teff and Tmem subset phenotypes (CD62L and CD27). (A and B) Concatenated contour plots with outliers and graphs showing the fraction of each Teff (CD127^−^) subset (Teff^early^, CD62L^hi^ CD27^+^; Teff^int^, CD62L^lo^ CD27^+^; and Teff^late^, CD62L^lo^ CD27^−^) (A) and Tmem (CD44^hi^ CD127^+^) subset (Tcm, CD62L^hi^ CD27^+^; Tem^early^, CD62L^lo^ CD27^+^; and Tem^late^, CD62L^lo^ CD27^−^) (B) that was BrdU^+^ at each time point. (C, D) Histograms showing percentages of CD11a^+^ BrdU^+^ Teff (C) and Tmem (D) subsets expressing *Ifng*/*Thy1.1*^+^. Error bars represent SEM. *, *P* < 0.05 for *Ifng*^+^ Tem^late^ compared to Tcm and Tem^early^; n.d., none detected.

In our previous work, we defined Teff and Tmem subsets of varying degrees of activation and differentiation, respectively, using the markers CD27 and CD62L. CD62L^lo^ Teff are short lived and make the most cytokines, while all Tmem subsets survive longer than the Teff (18). Teff^late^ (CD127^−^ CD62L^lo^ CD27^−^) are the terminal subset, with a substantial fraction showing signs of early apoptosis or death (18). With these functional differences in mind, we evaluated the maintenance of the subsets of Teff and Tmem derived from the peak of infection and their expression of *Ifng*. The proportions of Tmem precursor Teff (Teff^early^, CD62L^hi^ CD27^+^) and short-lived CD62L^lo^ Teff (SLEC), composed of intermediate Teff (Teff^int^, CD27^+^) and Teff^late^ (CD27^−^), out of the total BrdU^+^ CD11a^+^ CD4^+^ CD127^−^ Teff population was measured in the blood through 200 days p.i. (Fig. 2A). All subsets decayed dramatically between days 10 and 40 postinfection. Tmem (CD127^hi^ CD44^hi^) were also subdivided into central memory T cells (Tcm, CD62L^hi^ CD27^+^) and CD62L^lo^ Tem subsets (CD27^+^ Tem^early^ and CD27^−^ Tem^late^), which are listed here in the order of their level of differentiation (18). Tmem cells, surviving from the time of labeling during the peak of infection, maintained their proportions better than Teff over time, with cells in all Tmem populations remaining low but detectable on day 200 p.i. (Fig. 2B). Therefore, Plasmodium-responsive T cells with a memory phenotype do survive at physiological levels.

IFN-γ production by Th1 cells and *Ifng* BAC-In reporter expression can be stable after acute infection (19–24), but this is less well established in persistent infection, where exhaustion is common (25). In order to test for maintenance of *Ifng*, we observed the expression of the *Ifng*/*Thy1.1* Bac-In reporter on BrdU^+^ T cells in the blood through day 200. Despite this reporter including an RNA-stabilizing simian virus 40 (SV40) poly(A) tail, the *Ifng/Thy1.1* reporter became undetectable in all Teff subsets in the blood at day 80 p.i., while Tmem subsets were still detectable (Fig. 2C). Mature Teff (CD62L^lo^ Teff^int^ and Teff^late^) maintained a high and relatively constant fraction (18 to 30%) of *Ifng*/*Thy1.1*^+^ T cells from 10 to 30 days p.i., while Teff^early^ showed a transient peak of *Ifng* at day 10. In Tmem subsets, *Ifng*/*Thy1.1*^+^ cells were detected up to day 200 p.i. (Fig. 2D). The most differentiated subset of memory T cells, CD27^−^ Tem^late^, maintained the highest fraction of *Ifng*/*Thy1.1*^+^, significantly more than Tcm and Tem^early^ at three time points. Together, these data suggest that the fraction of *Ifng^+^* cells in CD62L^lo^ Teff subsets (Teff^int^ and Teff^late^) was quite constant until day 40, while highly differentiated Tem^late^ maintained constant *Ifng* expression through day 200. Notably, the disappearance of *Ifng^+^* Teff in blood by day 80 corresponds to the complete clearance of the parasite, which we have documented to occur between days 75 and 90 p.i. (7).

### Teff numbers and *Ifng* expression are promoted during premunition in the presence of parasite.

**FIG 3:**
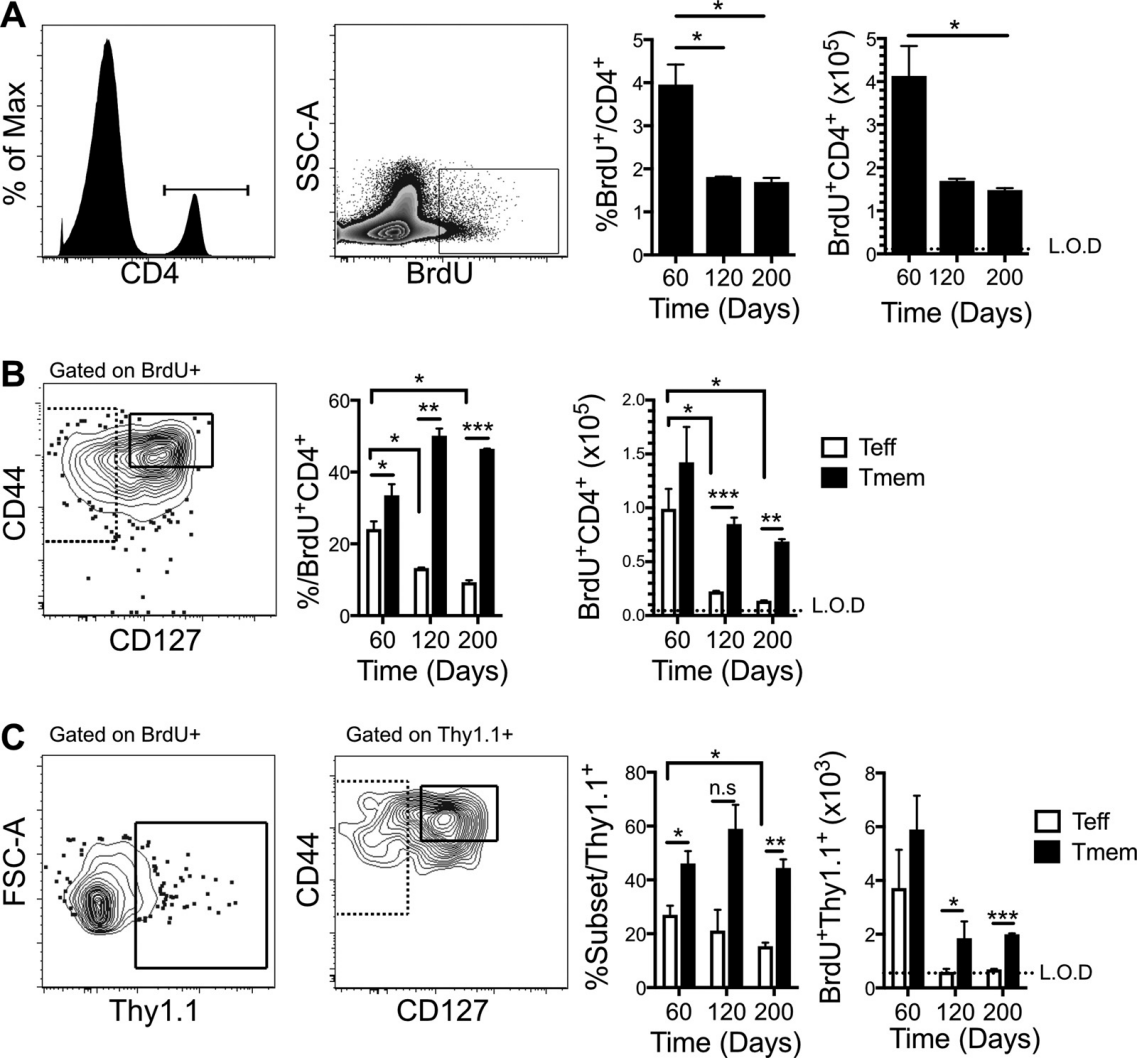
Decay of *Ifng*^+^ Teff between days 60 and 200 p.i. corresponds with decay of protection from heterologous infection. *Ifng*/*Thy1.1* BAC-In reporter mice were infected with P. chabaudi and given BrdU from days 3 to 10 p.i. to label dividing cells, and splenocytes were analyzed on days 60, 120, and 200 p.i. (A) Histogram and zebra plot show gating strategy, and graphs show percentages of splenic CD4 T cells that were still BrdU^+^ and numbers of BrdU^+^ CD4^+^ T cells recovered at each time point. SSC, side scatter. (B) Concatenated contour plot of BrdU^+^ T cells shows gating strategy, and graphs show the fractions of Teff (CD127^−^) and Tmem (CD44^hi^ CD127^hi^) out of BrdU^+^ CD4^+^ and the numbers of BrdU^+^ Teff and Tmem. (C) Contour plots show gating strategies for determining *Ifng*/*Thy1.1*^+^ out of BrdU^+^, and graphs show the fractions of BrdU^+^ *Ifng*/*Thy1.1*^+^ surviving as Teff and Tmem or total numbers of BrdU^+^ *Ifng*/*Thy1.1*^+^ Teff and Tmem. FSC, forward scatter. The limit of detection (L.O.D.) was calculated using all available data from uninfected animals from all experiments that did not receive BrdU. Data are representative of three experiments with five mice per group. Error bars represent SEM. The Student *t* test was used. *, *P* < 0.05; **, *P* < 0.01; ***, *P* < 0.001; n.s., not significant.

Loss of detectable *Ifng*^+^ Teff cells from the blood could represent their death, their migration into tissues, or their transition into Tmem. In this blood-borne infection, the tissue affected is the spleen. Therefore, to probe the migration and generation of Tmem from Teff cells that proliferated during the peak of infection, we quantified BrdU^+^ *Ifng^+^* Teff and Tmem in the spleen. Infected animals that received BrdU on days 3 to 10 p.i. had splenocytes harvested, and the proportions of BrdU^+^ cells out of CD4^+^ T cells and their numbers were measured on day 60 p.i., when premunition is detectable, day 120 p.i., when it is not, and day 200 p.i., when protection from parasitemia has been shown to be detectably lost (11). A decline of over half of the fraction and number of previously proliferated CD4 T cells occurred between days 60 and 120 (Fig. 3A). The decrease in total BrdU^+^ T cells from day 60 to day 120 is accounted for by the decrease in BrdU^+^ Teff numbers, not memory T cells (Fig. 3B). There were significant proportions and numbers of BrdU^+^ Tmem (CD127^hi^ CD44^hi^) and Teff (CD127^−^) above the threshold of detection in the spleen through day 200, but more Tmem at all of these late time points. Gating within *Ifng*/*Thy1.1*^+^ showed that the numbers of *Ifng^+^* Tmem in the spleen were also significantly higher than the numbers of *Ifng^+^* Teff on days 120 and 200 p.i., when *Ifng^+^* Teff disappeared to below the limit of detection (LOD) in the spleen (Fig. 3C). These data indicate that very few *Ifng*-expressing Teff derived from the peak of infection were maintained through day 120 after infection with P. chabaudi. Therefore, we hypothesized that T cells proliferating in response to the low-level persistent phase before day 60 contributed to the *Ifng*^+^ effector T cell population and to premunition, which fell off after day 60.

### Teff and Tmem differentiation and survival in chronic P. chabaudi infection.

**FIG 4:**
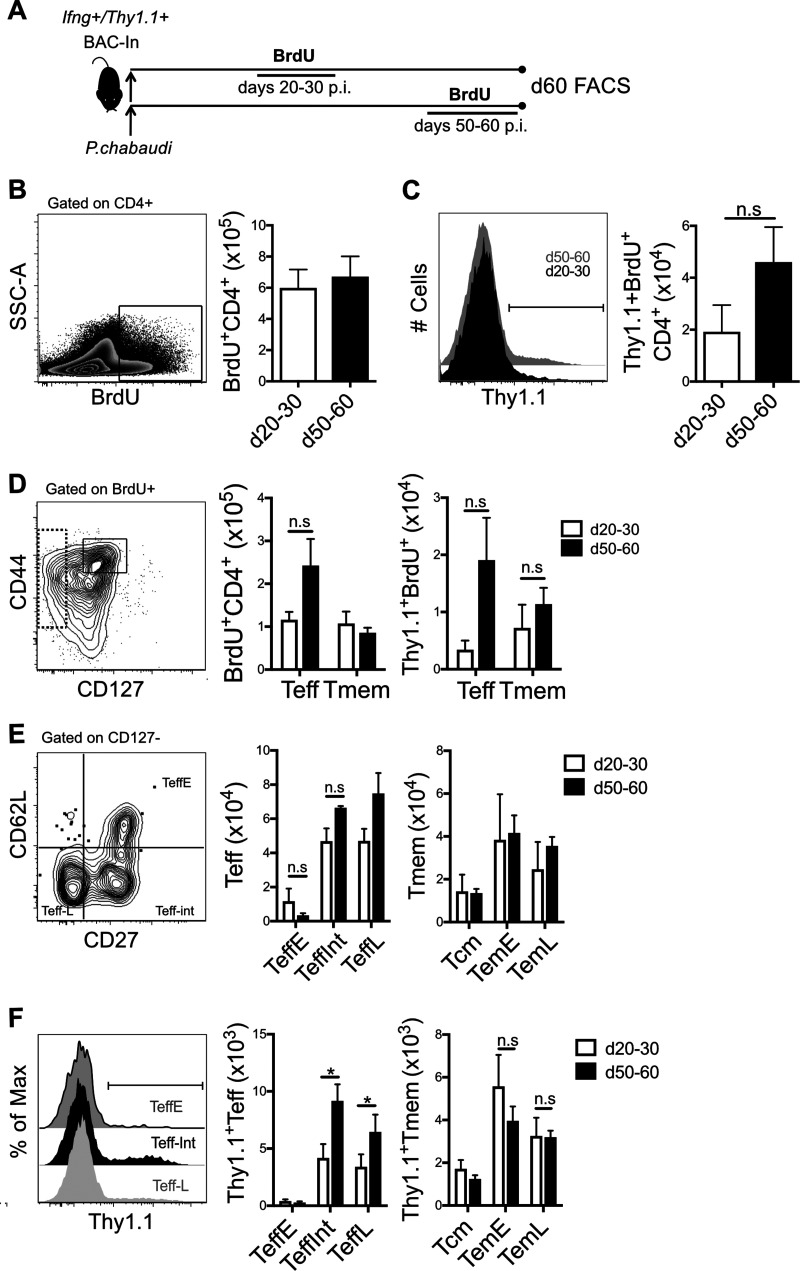
*Ifng*^+^ Teff are promoted by recent infection. (A) Schematic showing the experimental design. *Ifng*/*Thy1.1* BAC-In reporter mice were infected with P. chabaudi and given BrdU from days 20 to 30 or 50 to 60 p.i. Splenocytes were harvested at day 60 p.i. in both groups to study the day 60 phenotype of T cells generated during days 20 to 30 or 50 to 60 and their decay of *Ifng* expression. (B) Contour plot of BrdU^+^ out of CD4^+^ cells and graph of numbers of BrdU^+^ CD4^+^ cells generated within each time frame surviving until day 60. (C) Histogram showing representative example of *Ifng*/*Thy1.1*^+^ within BrdU^+^ CD4^+^ T cells and graph of the numbers of *Ifng*/*Thy1.1*^+^ BrdU^+^ CD4^+^ T cells generated with each time frame that were observed at day 60. (D) Gating strategy showing representative example of Tmem (CD44^hi^ CD127^hi^) Teff (CD127^−^) subsets from BrdU^+^ CD4^+^ T cells and graphs showing numbers of BrdU^+^ Teff and Tmem and *Ifng*/*Thy1.1*^+^ Teff and Tmem generated with each time frame that were observed at day 60. (E) Gating strategy showing representative example of Teff subsets to define early Teff (Teff^early^, CD62L^hi^ CD27^+^), intermediate Teff (Teff^int^, CD62L^lo^ CD27^+^), and late Teff (Teff^late^, CD62L^lo^ CD27^−^), as well as memory T cell subsets (not shown) including central memory T cells (Tcm, CD62L^hi^ CD27^+^) and effector memory T cells (CD62L^lo^ CD27^+^ Tem^early^ and CD62L^lo^ CD27^−^ Tem^late^). Graphs show numbers of cells in each of these subsets that were generated in each time frame of BrdU labeling and survived. (F) Histograms showing representative examples of *Ifng*/*Thy1.1*^+^ cells within subsets, and graphs showing the numbers of *Ifng*/*Thy1.1*^+^ cells generated during days 20 to 30 or 50 to 60 that had Teff and Tmem subset phenotypes on day 60. Error bars represent SEM. The Student *t* test was used. *, *P* < 0.05; n.s., not significant.

To investigate the possibility that continuous generation of effector T cells contributes to protection during premunition, we labeled T cells with BrdU *in vivo* at later time points, for 10 days starting either at day 20 or day 50 p.i., as shown in Fig. 4A, and studied the survival of T cells that had divided (BrdU^+^) before (days 20 to 30 p.i.) and after (days 50 to 60) parasitemia became undetectable by slide counting (LOD of <%0.001 infected red blood cells [iRBC] out of total RBCs). T cells were measured on day 60 p.i., when premunition was detectable. Interestingly, equal numbers of CD4^+^ BrdU^+^ (Fig. 4B) and *Ifng*/*Thy1.1*^+^ BrdU^+^ (Fig. 4C) T cells were observed at day 60, whether labeled on days 20 to 30 or on days 50 to 60 p.i. There was a trend toward more *Ifng*^+^ BrdU^+^ T cells in the more recently labeled group (days 50 to 60) than in those labeled a month prior; however, this did not reach significance. There was also a slight but nonsignificant increase in Teff cells and *Ifng*^+^ Teff, but not in Tmem, in more recently divided T cells (days 50 to 60) (Fig. 4D). Within the BrdU^+^ effector and memory T cells that were generated in the 10 days before either day 30 or day 60 p.i. and were still present at day 60 p.i., there were mostly CD62L^lo^ cells (Fig. 4E). There was a distinct and significant increase in *Ifng*/*Thy1.1*^+^ among mature Teff subsets that were more recently labeled (days 50 to 60) (Fig. 4F). These data support the conclusion that *Ifng* expression is maintained in mature Teff by recent stimulation, as first noted in adoptive transfer of reporter cells (16). In addition, the data suggest quite a short time frame for maintenance of *Ifng* accessibility in the absence of persistent infection, despite poly(A) tail-driven stabilization of the BAC-In *Ifng*/*Thy1.1* mRNA (20).

### IFN-γ in premunition and immune cell types mediating protection.

**FIG 5:**
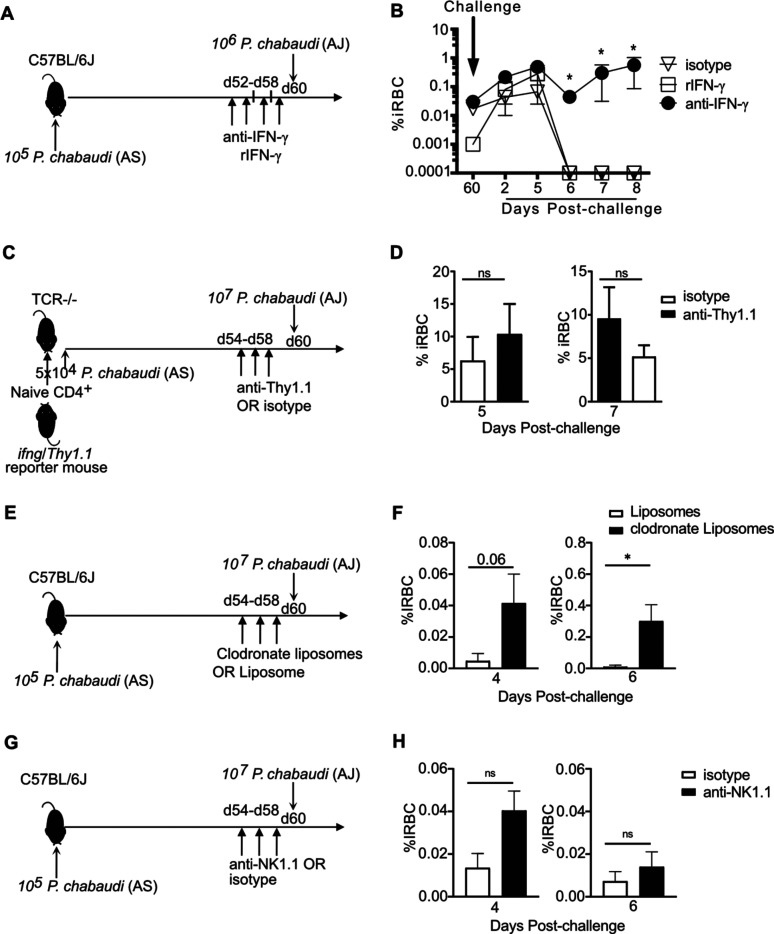
*In vivo* neutralization of IFN-γ prior to reinfection induces loss of premunition, and recombinant IFN-γ restores immunity to secondary infection. (A) Schematic showing the experimental design. C57BL/6 mice were infected with P. chabaudi, after which some animals were administered neutralizing anti-IFN-γ antibody or recombinant IFN-γ (rIFN-γ) every other day from day 52 to d60 p.i. Mice were then challenged with heterologous P. chabaudi AJ at day 60 post-primary infection. (B) Graph showing parasitemia measured at the indicated days postchallenge. (C) CD4 T cells from uninfected *Ifng*/*Thy1.1* knock-in mice were transferred into TCR^−/−^ mice, which were then infected with P. chabaudi AS. Some animals were administered anti-Thy1.1 antibody every other day from day 54 to day 58 p.i. Mice were then challenged with heterologous P. chabaudi AJ at day 60 post-primary infection. (D) Graphs showing parasitemia on days 5 and 7 of heterologous challenge of TCR^−/−^ animals. (E) C57BL/6 mice were infected with P. chabaudi. Some animals were administered clodronate liposomes or liposomes every day from day 55 to day 58 p.i. Mice were then challenged with heterologous P. chabaudi AJ at day 60 post-primary infection. (F) Graph showing parasitemia. (G) C57BL/6 mice were infected with P. chabaudi. Some animals were administered neutralizing anti-NK1.1 antibody every other day from day 54 to day 58 p.i. Mice were then challenged with heterologous P. chabaudi AJ at day 60 post-primary infection. (H) Graph showing parasitemia. Data are representative of five animals per group. Error bars represent SEM. The Student *t* test was used. *, *P* < 0.05; ns, not significant.

As the expression and decay of *Ifng* correlated with persistent infection, we tested the hypothesis that promotion of IFN-γ production by persistent infection is a mechanism for enhanced parasite immunity during chronic infection and investigated the relative importance of different potential IFN-γ-secreting cells in protection from reinfection during premunition. C57BL/6J mice were infected with P. chabaudi AS, and premunition to heterologous reinfection was tested on day 60 postinfection. Some infected animals (*n* = 5/group) were administered anti-IFN-γ neutralizing antibody or recombinant IFN-γ (rIFN-γ) or isotype over an 8-day period (days 52 to 60 p.i.) before heterologous challenge with P. chabaudi AJ at day 60 post-primary infection (Fig. 5A). Animals persistently infected with the AS clone were able to control heterologous challenge by day 6, suggesting that premunition had an effect on heterologous infection (Fig. 5B). We did not detect improvement of the excellent clearance observed in chronically infected mice upon pretreatment with rIFN-γ. However, if IFN-γ was blocked prior to reinfection during chronic infection, parasitemia was still uncontrolled, near 1.0%, after 8 days. Therefore, IFN-γ is essential to the protective effect induced by chronic infection known as premunition.

We next tested the hypothesis that *Ifng*^+^ T cells contribute to a state of premunition that exists before reinfection. In order to do this, we adoptively transferred naive CD4^+^ T cells from the *Ifng*/*Thy1.1* knock-in reporter mice into T cell receptor-deficient (TCR^−/−^) recipients. Recipient mice were then infected with P. chabaudi AS and treated with anti-Thy1.1 antibody during days 54 to 58 p.i. to deplete surviving *Ifng/Thy1.1*^+^ cells generated in the infection (Fig. 5C). *Ifng/Thy1.1*^+^ cells were completely depleted, as shown in Fig. S1 in the supplemental material; however, other T cells retained the ability to make IFN-γ quickly upon restimulation, as shown by intracellular cytokine staining. Parasitemia at days 5 and 7 of the second heterologous infection of TCR^−/−^ mice did not show a significant difference, but the interpretation is not clear. Either this result means that IFN-γ from nondepleted T cells controls parasitemia from reinfection or the critical IFN-γ does not come from T cells (Fig. 5D).

To test involvement of other cell types involved in Th1 immunity, we tested phagocytic cells and NK cells for a contribution to premunition. C57BL/6J mice were infected with P. chabaudi AS, followed by heterologous reinfection on day 60 postinfection. Some animals from each group were administered either clodronate liposomes for phagocyte depletion, as diagrammed (Fig. 5E) or anti-NK1.1 antibody for NK cell depletion (Fig. 5G) before heterologous challenge. The mice treated with clodronate liposomes before reinfection had significantly higher parasitemia than control mice treated with liposomes (Fig. 5F). In contrast, NK cell depletion (Fig. S2) before reinfection did not significantly affect the parasitemia (Fig. 5H). These data indicate that both IFN-γ and phagocytes are involved in the immune mechanism mediating protection from reinfection during the persistent phase of P. chabaudi infection.

### Persistent infection decreases memory T cell numbers but maintains *Ifng*^+^ Teff.

**FIG 6:**
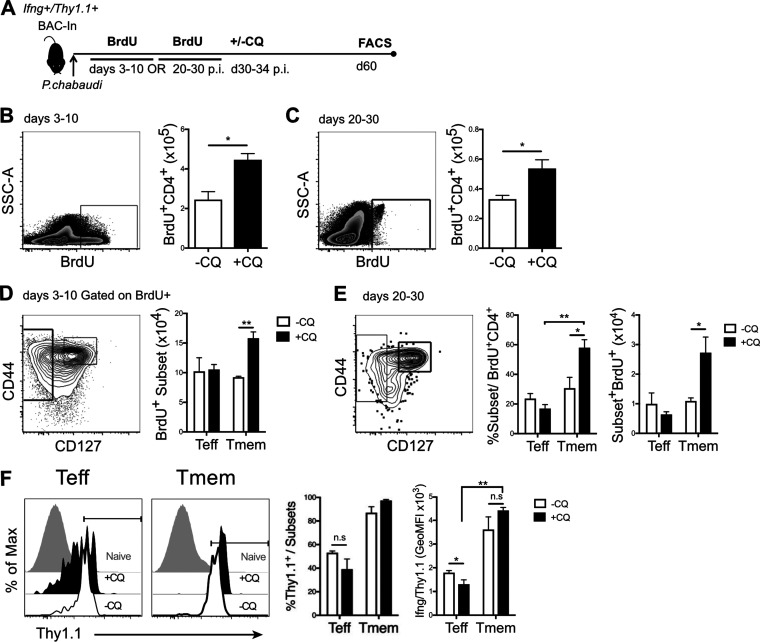
Chronic infection impacts Tmem survival but enhances *Ifng* expression. (A) Schematic showing the experimental design. *Ifng*/*Thy1.1* BAC-In reporter mice were infected with P. chabaudi and given BrdU from days 3 to 10 or 20 to 30 p.i. Some animals were treated with chloroquine (+CQ; days 30 to 34 p.i.) to clear residual parasitemia. Splenocytes were harvested at day 60 p.i. for flow cytometry analysis of survival and *Ifng*/*Thy1.1* expression. (B and C) Zebra plots and graphs showing numbers of BrdU^+^ CD4^+^ T cells in animals receiving BrdU during days 3 to 10 (B) or days 20 to 30 (C) p.i. in +CQ and −CQ groups. (D and E) Contour plots and graphs showing fractions of Teff (CD127^−^) and Tmem (CD44^hi^ CD127^hi^) out of BrdU^+^ CD4 T cells and numbers of total BrdU^+^ Teff and Tmem in animals receiving BrdU during days 3 to 10 (D) or days 20 to 30 p.i. (E) in +CQ and −CQ groups. (F) Histograms and graphs showing the fractions of *Ifng*/*Thy1.1*^+^ cells within and the levels of *Ifng*/*Thy1.1* expression on BrdU^+^ Teff and Tmem in +CQ and −CQ control groups. GeoMFI, geometric mean fluorescence intensity. Error bars represent SEM. The Student *t* test was used. *, *P* < 0.05; **, *P* < 0.01; n.s., not significant.

In order to test the effect of persistent parasites on the maintenance of T cell numbers and *Ifng* accessibility, residual parasitemia was cleared using an antimalarial drug before measurements. To label Plasmodium-specific T cells, *in vivo* BrdU labeling was performed (days 3 to 10 or 20 to 30 p.i.) in infected IFN-γ reporter mice, followed by elimination of persistent parasitemia using chloroquine (CQ) treatment (days 30 to 34 p.i.) (Fig. 6A). Surviving BrdU^+^ T cells were detected on day 60 postinfection. Persistently infected non-CQ-treated (−CQ) animals had significantly fewer surviving BrdU^+^ CD4^+^ T cells at day 60 p.i. than did persistently infected but chloroquine-treated (+CQ) animals (Fig. 6B). We also tested the effect of the large initial peak of parasitemia on Tmem survival. However, the results were similar whether we tracked T cells that had proliferated during the first peak of T cells (days 3 to 10) or during a period (days 20 to 30) when parasitemia was controlled to below a still detectable 1% (7) and less T cell proliferation had occurred (Fig. 6C). Although there were roughly equal fractions and numbers of Teff and Tmem surviving in the −CQ group, the +CQ group showed a significantly larger Tmem (CD127^hi^) than Teff (CD127^−^) population (Fig. 6D). The increased presence of Tmem at day 60 also occurred in +CQ animals when the Tmem precursor cells had been generated during days 20 to 30 p.i. (Fig. 6E). The fraction of *Ifng^+^* Tmem was greater than the fraction of *Ifng*^+^ cells within Teff (Fig. 6F). *Ifng* expression per cell (as measured by geometric mean fluorescence intensity) (Fig. 6F, far right) was higher in Teff, but not Tmem, in persistently infected animals (−CQ). Together, these data support and extend previous reports that chronic infection reduces the survival of CD4 memory T cells (26) and that chronic infection maintains cytokine expression in response to P. chabaudi (16), specifically *Ifng* at the transcriptional level in effector T cells. Therefore, we determined to look at a larger group of Th1 cytokines.

### Persistent infection promotes IFN-γ^+^ TNF^+^ IL-2^−^ T cells.

**FIG 7:**
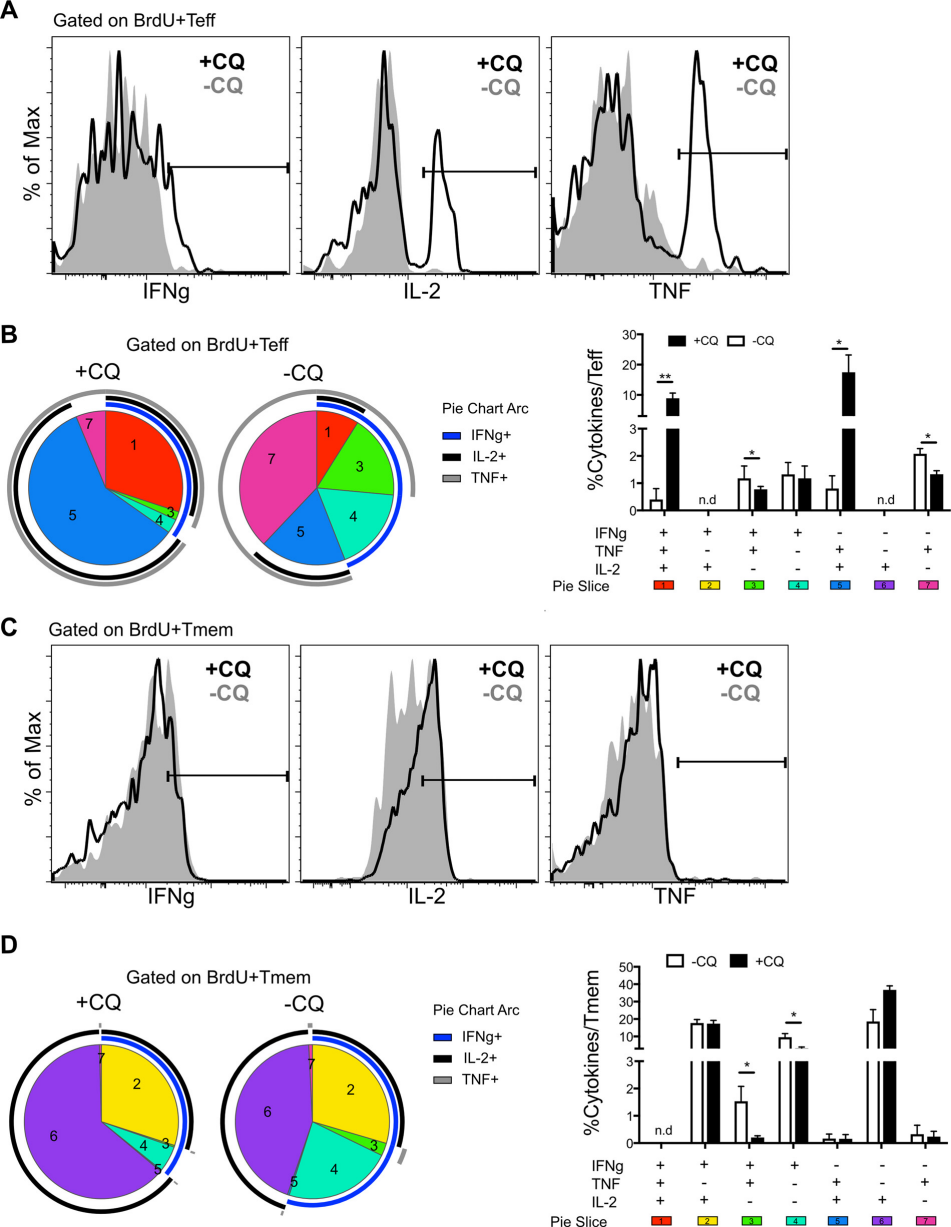
Tmem make the highest cytokine levels, and persistent infection promotes IFN-γ^+^ TNF^+^ IL-2^−^ T cells, though it reduces Teff multicytokine producers. *Ifng*/*Thy1.1* BAC-In reporter mice were infected with P. chabaudi and given BrdU from days 20 to 30 p.i. Some groups were treated with chloroquine (+CQ; days 30 to 34 p.i.) to cure residual parasitemia. Splenocytes were harvested at day 60 p.i. for intracellular cytokine staining. (A and C) Histograms showing the fractions of IFN-*γ*, IL-2, and TNF production in Teff (A) and Tmem (C) in +CQ (dark line) and −CQ (gray line) mice. (B and D) Pie charts with slices representing combinations of markers and bar graphs showing Boolean gating analysis of all possible combinations of IFN-*γ*, IL-2, and TNF production within Teff (B) and Tmem (D) in +CQ (black bars) and −CQ (white bars) mice. Arc lines around the pie graphs represent the fractions of IFN-γ^+^ (blue), IL-2^+^ (black), and TNF^+^ (gray) cells. Data are representative of three independent experiments with four animals per group. Error bars represent SEM. The Student *t* test was used. *, *P* < 0.05; **, *P* < 0.01; n.d., none detected.

To identify the cytokines produced by effector and memory T cells surviving into the phase of premunition and their dependence on persistent parasitemia, we measured IFN-γ, TNF, and IL-2 production by BrdU^+^ T cells in the spleen using intracellular cytokine staining after BrdU labeling from day 20 to day 30 (Fig. 5C and E). Gating on CD127^−^ BrdU^+^ Teff, it was clear that Teff cytokine production upon restimulation was severely limited in persistent infection (Fig. 7A). Despite *in vivo Ifng* expression, there was only a slight shift in IFN-γ intracellular staining, with clear peaks for TNF and IL-2 only in the drug-treated group. Boolean gating analysis of the distribution of cytokines produced by each Teff population is represented in pie charts and histograms (Fig. 7B). Teff in persistent infection had significantly lower fractions of triple- and double-cytokine producers (IFN-γ^+^ TNF^+^ IL-2^+^ and IFN-γ^−^ TNF^+^ IL-2^+^) than +CQ Teff. In contrast, BrdU^+^ Tmem from persistent infection exhibited significant IFN-γ and IL-2 intracellular cytokine staining above the background (Fig. 7C). Qualitative differences in the cytokine combinations expressed in Teff and Tmem were also noted. IL-2 single producers and IFN-γ^+^ IL-2^+^ TNF^−^ were detected in Tmem but not in Teff. Single producers of IFN-γ were higher in Tmem than in Teff, and these cells were also slightly and significantly increased in −CQ Tmem. The higher IFN-γ protein levels in Tmem (Fig. 7D) compared to the levels in Teff (Fig. 7A) confirm the results seen in the transcriptional readout from the *Ifng* reporter presented in Fig. 6F.

The finding that correlates with the protection maintained by persistent infection, i.e., in −CQ animals compared to treated mice (+CQ), is that IFN-γ^+^ TNF^+^ IL-2^−^ cells and IFN-γ single producers were significantly increased among Tmem in persistently infected animals compared to their levels in treated mice. In previous work, a CD62L^lo^ IFN-γ^+^ TNF^+^ IL-2^−^ population was also detected as increased in MSP-1 specific TCR transgenic (Tg) T cells from chronically infected animals compared to the population in treated animals at day 60 p.i. (14). This interesting subset appeared to be present within both CD127^−^ Teff and CD127^hi^CD44^hi^ Tmem.

## DISCUSSION

In this study, we studied two time periods of declining protection from Plasmodium infection. In addition to loss of premunition as parasites disappear after a few months, protection wanes again measurably by day 200 p.i. (27). T cells have been shown to be affected by persistent infection more than by antibody during both periods (11, 14). Our data add to these previous studies and flesh out our understanding of the effect of persistent infection on T cells, both as the parasite declines and then later, as the T cell response diminishes enough for parasites to be able to grow again.

Overall, our findings support the interpretation that the promotion of *Ifng^+^* Teff by chronic infection enhances immunity. In the later time period, we show that *Ifng*^+^ T cells in the spleen decay precipitously once the period of premunition is over and then Teff become undetectable, even in the spleen, by day 200, coinciding with the second reduction in protection by day 200 (11). The kinetics of decay of Teff numbers after day 60, in combination with our previous observation that Teff from P. chabaudi infection protect better than Tmem, also indicate that the Teff decline is likely the cause of loss of immunity after day 60. Recombinant IFN-γ administered systemically in the days before reinfection has been shown to enhance long-term protection in P. chabaudi infection, specifically promoting sterile immunity upon reinfection at day 200 (28, 29). Our previous work and that of others shows that there are few if any fully Th1-committed (T-bet^hi^) T cells generated in P. chabaudi infection (16, 30, 31). For example, 94% of *Ifng*/*Thy1.1*^+^ BAC-In Teff transferred from infected donors (day 8 p.i.) to infection-matched recipients lose *Ifng* accessibility (expression of BAC-In *Ifng*/*Thy1.1*) by day 60 (16). Importantly, the disappearance of Teff detectable in the blood by day 200 and *Ifng*^+^ Teff by day 100 p.i. corresponds to the time frame when loss of protection, or reappearance of susceptibility to P. chabaudi parasitemia upon reinfection, was documented by Freitas do Rosario et al., between day 120 and day 200 p.i. (11). Together, these three studies suggest that decay of *Ifng*^+^ Teff explains the second decay of protection from microscopically detectable reinfection, but not premunition.

In addition to improving our understanding of the long-term decay of immunity, this study identifies some changes in T cell function specifically downstream from low-level persistence of parasites. The literature suggests that while maintenance of antigen-specific T cells after stimulation does not require major histocompatibility complex class II (MHC-II), survival and protective CD4 T cell functions can be improved by persistent antigen (32–34). In addition, while resting Tcm are capable of providing some long-term immunity against Leishmania major, a prototypical persistent pathogen, persistence of the pathogen actually promotes solid immunity to reinfection (35). However, features of persistent stimulation that contribute to protection have been elusive. Multifunctional Th1 cells (IFN-γ^+^ TNF^+^ IL-2^+^) have been shown to correlate with protection after vaccination against Leishmania (36), though it has not been established if these are long-lived Tmem or short-lived Teff. Our work suggests that in cured persistent infection, these cells are largely Teff. As chronic infection enhances protection against both Leishmania and Plasmodium (7, 37), T cell multifunctionality, protective in *Leishmania*, could be the correlate of immunity during premunition (15). However, persistently stimulated (−CQ) Teff have a significantly lower fraction of triple-cytokine producers (IFN-γ^+^ TNF^+^ IL-2^+^) than rested T cells (+CQ). On the other hand, an IFN-γ^+^ TNF^+^ IL-2^−^ CD127^+/−^ T cell subset has been shown, using an adoptive transfer system, to be increased in persistent P. chabaudi infection compared to its proportion in treated animals (14). Here, we show that in the polyclonal response, these IFN-γ^+^ T cells are also promoted by persistence of parasites and that these cells contain both Teff and Tmem. Both intracellular cytokine staining and reporter data show that more Tmem than Teff produce cytokines; however, triple-cytokine producers on day 60 are confined to BrdU^+^ CD127^−^ Teff. This surprising result suggests that triple-cytokine-producing T cells, reported to correspond to protection in several models, are short-lived effector T cells. CD127 expression and longevity will have to be tested in other models to confirm that this is true in other infections. However, multifunctional T cells are also curtailed by persistent infection, at least when measured by intracellular cytokine staining, despite persistent infection being protective, suggesting that multifunctional-cytokine-producing CD4 T cells are not a correlate of protection in the case of premunition.

In order to determine the role of IFN-γ in the first phase of protection, where *Ifng*^+^ Teff are promoted by persistent infection, we tested the effect of neutralizing IFN-γ on the premunition phase. Neutralization of IFN-γ before infection eliminated the improved protection provided by persistent infection, or premunition. In addition, we tested the contribution of NK cells and phagocytic cells to protection in this phase. Depletion of phagocytic cells, but not NK cells, also reduced the effect of premunition and prolonged the parasitemia after the reinfection challenge. Each of these cell types may contribute to IFN-γ production. NK cells and phagocytes can contribute, for example, in Salmonella infection (38). Neutrophils and microglia have measurable effects on Toxoplasma infection (39) *in vivo*. While training of phagocytes is reported to occur by persistent viral infection (40), it is challenging to determine from these experiments if Th1 cytokines associated with persistent infection are primarily critical in the period before reinfection, for training, or also during reinfection. Each of the experiments one could envision for this test has caveats, which is why we performed several. Both antibodies and clodronate have the potential to persist for weeks, and it would be difficult to establish the threshold at which they are no longer potent. It is also possible that the antigen presentation and Th1 response are reduced after clodronate liposome treatment. Therefore, the limited conclusion is that Th1 type responses, and not just antibody, are critical for rapid clearance of repeat infection during persistent infection. This assertion is also supported by our recent demonstration that STAT3 deficiency in T cells shifts the response toward Th1 and protects in Plasmodium reinfection, (30). In addition, da Silva et al. show that recombinant IFN-γ can promote heterologous protection once T cell immunity decays at day 200 p.i., supporting a role for a Th1-type response in protection (29). It will be important to use technology to attempt to discriminate between the effects of innate cell conditioning during premunition and adaptive cell memory reactivation upon reinfection.

Here, we also showed in a physiological setting that Tmem survive and contain a population of Tem that maintain *Ifng* expression. We and others have shown that this CD27^−^ population is propagated by the other populations (14, 41). This confirms and extends our previous work, using P. chabaudi MSP1-specific B5 TCR Tg, that showed that all Tmem subsets, including Tem, survive longer than Teff (18). However, there is definitely an effect of persistent infection on Tmem numbers. This could be interpreted as a deficiency in Tmem in chronic infection, except that immunity to reinfection is actually better in low-level persistent infection than after cure of the same infection (5). Similar experiments have been done using naive adoptively transferred B5 TCR Tg Teff and have showed a similar decrease in Tmem in persistently infected mice compared to treated mice (14). Our previous work on mechanisms of Tem survival shows that, in addition to maintenance of Tem numbers in the absence of antigen, adoptively transferred Tem can proliferate in response to the low-level persistent infection late in P. chabaudi infection, though only the poorly protective CD27^+^ Tem^early^ subset expand in number as a result (15). Therefore, the present data suggest that persistent infection promotes *Ifng* persistence in short-lived *Ifng*^+^ Teff, but not Tem, and that this constitutes an important facet of premunition.

## MATERIALS AND METHODS

### Mice and infections.

*Ifng*/*Thy1.1* BAC-In and *Ifng*/*Thy1.1* knock-in mice were a kind gift from Casey Weaver (University of Alabama, Birmingham, AL). C57BL/6 and TCRβ/δ^−/−^ (B6.129P2-Tcrb^tm1Mom^ Tcrd^tm1Mom^) mice were purchased from the Jackson Laboratory (Bar Harbor, ME). The BAC-In *Ifng*/*Thy1.1* construct is a faithful marker of the accessibility of the *Ifng* locus, representing a fraction of the total cells that stain positive for IFN-γ protein using intracellular cytokine staining (20). The construct includes an SV40 poly(A) tail to stabilize the reporter at the RNA level, so expression has been shown to last at least 40 days after its first expression (20). These mice were maintained in our specific-pathogen-free animal facility with *ad libitum* access to food and water. Mice 6 to 12 weeks old were infected intraperitoneally (i.p.) with 10^5^ Plasmodium chabaudi *chabaudi* AS (courtesy of Jean Langhorne, Francis Crick Institute, London, UK). For protection assays, C57BL/6 mice were infected i.p. with 10^5^ P. chabaudi AS and challenged with 10^6^ P. chabaudi AJ (MR4, Manassas, VA)-infected erythrocytes. P. chabaudi was maintained as described elsewhere (7, 42). Parasites were counted by light microscopy in thin blood smears stained with Giemsa stain (Sigma-Aldrich, St. Louis, MO). All animal studies were carried out in accordance with the protocol as approved by the University of Texas Medical Branch Institutional Animal Care and Use Committee.

### *In vivo* assays.

For *in vivo* labeling with 5-bromo-2′-deoxyuridine (BrdU; Sigma), BrdU was given in drinking water (0.8 mg/mL) on days 3 to 10 or 20 to 30 postinfection (p.i.). In some experiments, mice were treated with a dose of 50 mg/kg of body weight of the antimalarial drug chloroquine (CQ) i.p. on days 30 to 34 postinfection. C57BL/6 mice were infected with P. chabaudi and then treated with four 0.5-mg/mouse doses of depleting monoclonal antibodies (MAbs) against IFN-γ or isotype control antibody (H22; Thermo Fisher Scientific, Waltham, MA) or four 15-ng/mouse doses of recombinant mouse IFN-γ (rIFN-γ expressed in E. coli; PeproTech, Rocky Hill, NJ) i.p. every 2 days starting 52 days p.i., as previously established (29). For NK cell depletion, mice were injected with either an anti-NK1.1 antibody or an isotype control antibody (PK136; BioXCell, Lebanon, NH). Clodronate liposomes have been used for phagocytic cell depletion in several immunology studies to investigate their functions (43, 44). Therefore, 5 doses per day of 100 μL of clodronate liposomes (0.5 mg) or liposomes were administered to chronically infected mice starting 55 days before reinfection.

In some experiments, *Ifng*/*Thy1.1* CD4 T cells from uninfected 5-week-old animals were purified by magnetically activated cell sorting (MACS) and transferred into TCRβ/δ^−/−^ (TCR^−/−^) recipients, which were infected and then treated three times with anti-Thy1.1 antibody (250 μg/mouse i.p.) (clone 19E12; BioXCell, West Lebanon, NH) every other day between days 56 and 60 p.i., similar to the experiment described in reference 45.

### Flow cytometry.

Blood was collected by tail bleeding with a heparin-coated pipette tip and incubated in RBC fix/lysis buffer (eBioscience, San Diego, CA) for 10 min at room temperature before washing with phosphate-buffered saline (PBS) containing 2% fetal bovine serum (FBS) and 0.1% sodium azide (fluorescence-activated cell sorting [FACS] buffer; Sigma-Aldrich, St. Louis, MO). Cells were then stained with anti-CD90.1 antibody (30-H12), anti-CD44 antibody (IM7), and anti-CD27 antibody (LG-7F9) and the following combinations of fluorophores peridinin chlorophyll protein (PerCP)/efluor710, allophycocyanin (APC)/efluor780, and APC-conjugated antibodies (all from eBioscience) and anti-CD127 (A7R34)-antibody phycoerythrin (PE)-cyanine-5, anti-CD62L antibody (MEL-14)-brilliant violet 605, and anti-CD4 (RM4-5) antibody-brilliant violet 650 (BioLegend, San Diego, CA).

Single-cell suspensions from spleens were prepared in HEPES-buffered Hanks balanced salt solution (HBSS; Life Technologies, Thermo Fisher Scientific, Waltham, MA) and prepared with RBC lysis buffer (eBioscience) before washing with FACS buffer. Cells were then stained with Fc receptor blocking antibody (clone 2.4G2; BioXCell), followed by surface staining as described above for blood. Cells were stained using the fluorescein isothiocyanate (FITC) BrdU flow kit (B44, BDbiosciences) and analyzed by flow cytometry according to the manufacturer’s protocol.

For intracellular staining, splenocytes (5 × 10^6^ per mL) were stimulated with phorbol myristate acetate (PMA) (50 ng/mL) and ionomycin (500 ng/mL) at 37°C for 5 h in complete Iscove’s modified Dulbecco’s medium (cIMDM). Brefeldin A (10 μg/mL) was added for the last 2 h (all from Sigma). Cells were processed for surface staining as described above and fixed with 2% paraformaldehyde (Sigma-Aldrich), followed by permeabilization using 1× permeabilization buffer (BD Biosciences). Cells were washed 3 times in permeabilization buffer and incubated for 40 min with anti-IFN-γ antibody (XMG1.2)-FITC, anti-IL-2 antibody (JES6-5H4)-CF594, and anti-TNF antibody (MP6-XT22)-PE/Cy7 or isotype controls (all from eBioscience). Cells were collected on an LSRII Fortessa using FACSDiva software (BD Biosciences) and analyzed in FlowJo (version 9.7; Tree Star, Ashland, OR). Compensation was performed in FlowJo using single-stained splenocytes (with CD4 in all colors). Boolean gating analysis was performed, and the distribution of cytokines was analyzed with Spice version 6 (46) and Prism (version 7; GraphPad, La Jolla, CA). The pie charts in Fig. 7 show fractions of cytokine producers only. Data from each mouse were analyzed, and average values and standard errors of the means (SEM) were calculated for graphs and contour plots. Data from five mice were concatenated to achieve sufficient cell numbers for presentation.

### Statistical analysis.

All data are presented as mean values ± SEM. The two-tailed unpaired Student *t* test was used (Prism; GraphPad, La Jolla, CA). The limit of detection (LOD) was calculated using all available data from animals from all experiments that did not receive BrdU and is defined as the mean of the background values times 1.645.
