# Supplementary material for: Effects of Low-Level Persistent Infection on Maintenance of Immunity by CD4 T Cell Subsets and Th1 Cytokines
Source: Infect Immun. 2023 Feb 28;91(3):e00531-22. doi: 10.1128/iai.00531-22 (PMC10016079; doi:10.1128/iai.00531-22)
Supplement: Supplemental file 1 — Fig. S1 and S2. Download iai.00531-22-s0001.pdf, PDF file, 1.2 MB [file iai.00531-22-s0001.pdf]

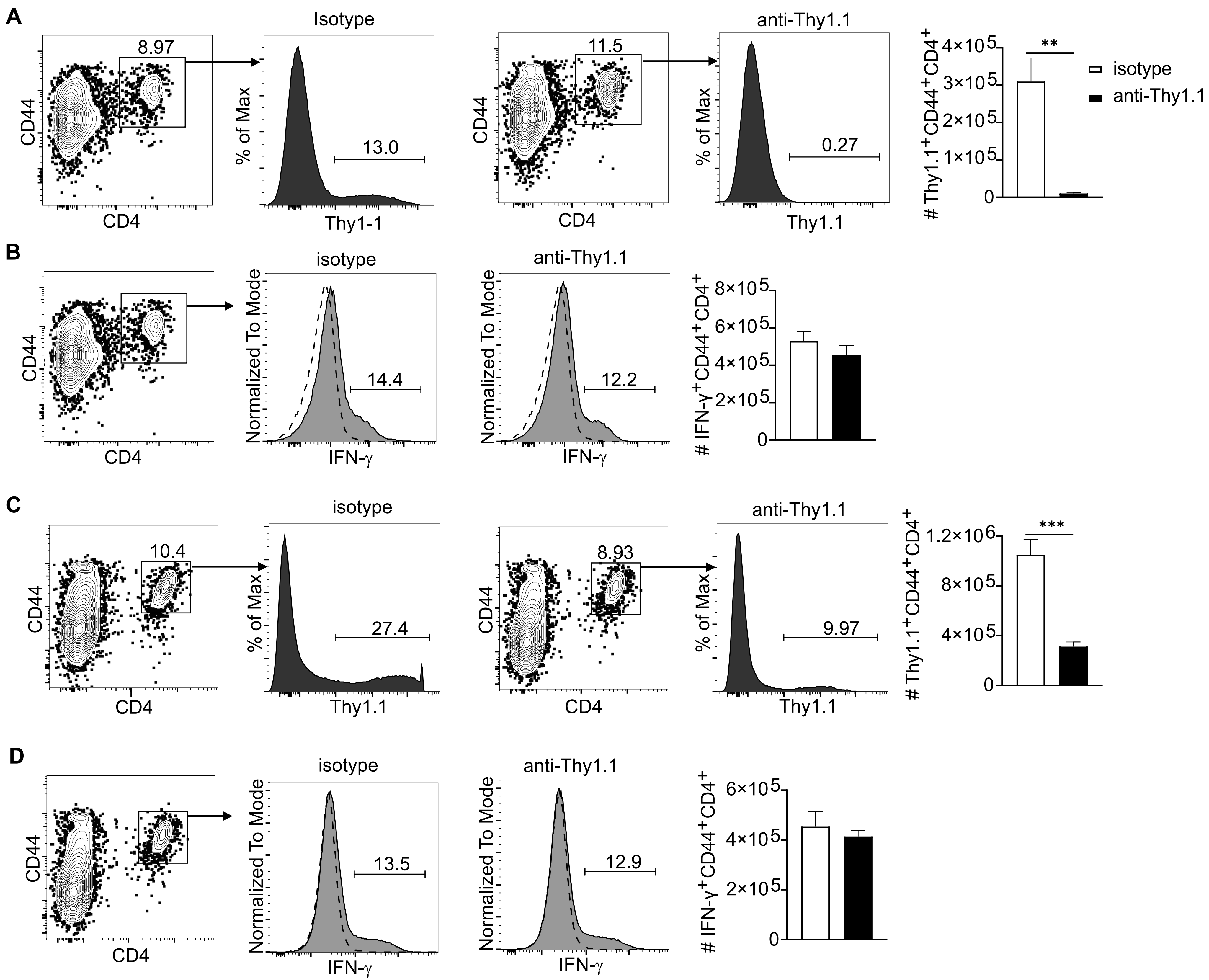
A
Isotype
anti-Thy1.1
8.97
11.5
CD44
CD4
% of Max
13.0
0.27
Thy1-1
Thy1.1
Thy1.1+CD44+CD4+
4×10⁵
3×10⁵
2×10⁵
1×10⁵
0
**
isotype
anti-Thy1.1
B
isotype
anti-Thy1.1
Normalized To Mode
14.4
12.2
IFN-γ
IFN-γ+CD44+CD4+
8×10⁵
6×10⁵
4×10⁵
2×10⁵
C
isotype
anti-Thy1.1
10.4
8.93
27.4
9.97
Thy1.1
1.2×10⁶
8×10⁵
4×10⁵
***
D
isotype
anti-Thy1.1
13.5
12.9
6×10⁵
4×10⁵
2×10⁵

Supplemental Figure 1: Depletion of *Ifng*-reporter cells and IFN-γ production after stimulation

CD4 T cells from uninfected *Ifng*/Thy1.1 Knock-In mice were transferred into $TCR^{-/-}$ mice, which were then infected with *P. chabaudi* AS. Some animals were administered anti-Thy1.1, every other day from day 54 to d58 p.i.. Splenocytes were harvested at day 60 p.i. or day 20 post challenge for flow cytometry analysis to check the depletion *Ifng*/Thy1.1 Knock-In and for intracellular cytokine staining. Plots, Histograms and graph showing gating and the fraction of (**A**) *Ifng*/Thy1.1 Knock-In cells in $CD4^{+}CD44^{hi}$ after depletion at day 60 p.i.; (**B**) $CD4^{+}CD44^{hi}$ gated IFN-γ intracellular cytokine staining at day 60 p.i.; (**C**) *Ifng*/Thy1.1 Knock-In cells in $CD4^{+}CD44^{hi}$ after challenge at day 20 p.c.; (**D**) $CD4^{+}CD44^{hi}$ gated IFN-γ intracellular cytokine staining after challenge at day 20 p.c., dashed line represents isotype control for intracellular staining. Data are representative of three animals per group. Error bars represent SEM. **p < 0.01, ***p < 0.001 Student's *t* test.

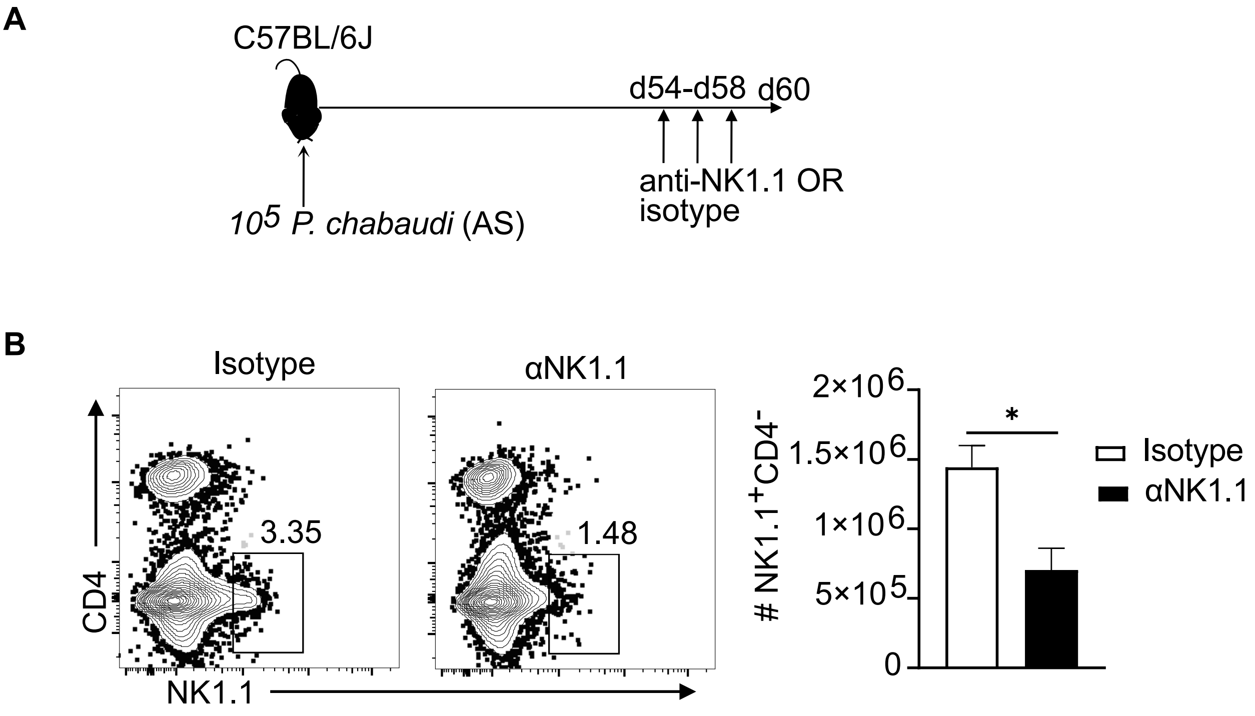

Supplemental Figure 2. Depletion of NK cells

(A) C57BL/6 mice were infected with *P. chabaudi*. Some animals were administered neutralizing anti-NK1.1 every other day from day 54 to d58 p.i.. Splenocytes were harvested at day 60 p.i. for flow cytometry analysis to check NK cell depletion. (B) Plots and graph showing gating the fraction and the number of NK cells after depletion. Data are representative of three animals per group. Error bars represent SEM. *$p < 0.05$ Student's *t* test.
